# Half-jumping plant lice – a taxonomic revision of the distinctive psyllid genus *Togepsylla* Kuwayama with a reassessment of morphology (Hemiptera, Psylloidea)

**DOI:** 10.3897/zookeys.716.13916

**Published:** 2017-11-27

**Authors:** Xinyu Luo, Wanzhi Cai, Gexia Qiao

**Affiliations:** 1 Key Laboratory of Zoological Systematics and Evolution, Institute of Zoology, Chinese Academy of Sciences, No. 1 Beichen West Road, Chaoyang District, Beijing 100101, China; 2 Department of Entomology, China Agricultural University, No. 2 Yuanmingyuan West Road, Haidian District, Beijing 100193, China; 3 College of Life Sciences, University of Chinese Academy of Sciences, Beijing 100049, China

**Keywords:** Aphalaridae, morphological characters, Oriental region, Togepsyllinae

## Abstract

Togepsyllinae is a distinctive group within Psylloidea, with its systematic status treated variously by different authors. Of the only two known genera, *Togepsylla* is better known and distributed in temperate and tropical East Asia. In this study, the taxonomy and morphology of *Togepsylla* was studied in detail. Keys to adults and fifth instar immatures of the genus are provided. *Togepsylla glutinosae* **sp. n.** is described, and *T. matsumurana*, *T. takahashii*, and *T. tibetana* are redescribed. *Syncoptozus* is compared with *Togepsylla* for differences in morphology. Modern psyllids have evolved their jumping hind legs via the elongation of the dorsal edge of coxa, the broadening of coxal wall, the thickening of meron, the backward twisting of the plane of trochanter, femur, and tibia, and the enlargement of trochanteral tendon. However, in *Togepsylla*, this modification has progressed halfway. The metapleuron of *Togepsylla* is arranged in a different way than other psyllids. The pleural sulcus is short, and the metepisternum and trochantin are not divided. Wax-secreting fields on abdominal sternites, resembling those of whiteflies, are found on all *Togepsylla* species, and described for the first time. Other distinctive characters of the genus are also revealed, e.g. frons completely fused with gena, a pair of extra sclerites present behind the base of thoracic furca, one-segmented aedeagus, and absence of a flag lobe on valvula dorsalis of ovipositor. Based on various similarities in morphology, Togepsyllinae may have a close relationship with Aphalaridae-Rhinocolinae and is possibly related to Homotomidae, Liviidae-Liviinae and *Atmetocranium* (Calophyidae). All the distinctive characters of Togepsyllinae suggest that the current placement of the group is doubtful, and the phylogeny of Aphalaridae needs to be resolved.

## Introduction

Psylloidea, the superfamily of jumping plant lice, is a group of phloem-sucking insects with strict host specificity. Among all its subordinate taxa, the Oriental and Palaearctic genus *Togepsylla* is undoubtedly one of the most distinctive. Named after the symmetrical and articulated thick ‘spines’ on the dorsum of head and thorax, it is readily diagnosed by these spine-like setae and wings held flat over its dorsum instead of roof-wise, in addition to the more intrinsic characters identified in this article. The genus contains only three known species to date: *Togepsylla matsumurana* Kuwayama, 1949, *T. takahashii* Kuwayama, 1931 (type species), and *T. tibetana* (Yang & Li, 1981). The few known species develop exclusively on Lauraceae plants, often inducing pit galls or leaf-rolling galls (Takahashi 1936; Miyatake 1970; Li 2011), in contrast to their spine-lacking Neotropical relative *Syncoptozus* Enderlein, which develops on plants of Magnoliaceae. The two genera compose the small taxon Togepsyllinae/-ini with valid monophyly. Yang and Li (1981) erected *Hemipteripsylla* to contain *H. tibetana* and *T. matsumurana*, which possess relatively short setae based on short projections and lack tiny spines on the dorsum of aedeagus. These characters were accurately recognized, although considered insufficient to support an alternative genus, by Hodkinson (1990), who synonymized *Hemipteripsylla* with *Togepsylla*.

Togepsyllini was established by Bekker-Migdisova (1973), who assigned it to her sense of Carsidaridae-Tenaphalarinae. Before Bekker-Migdisova, Kuwayama (1931) placed *Togepsylla* in Carsidarinae, whereas Miyatake (1970) moved the genus into Pauropsyllinae. White and Hodkinson (1985) presented the first calculated cladogram of Psylloidea, using a numerical method. They treated the group as Togepsyllinae, assigning it in Aphalaridae, one of the eight families recognized in their classification. The authors postulated an extinct Rutales-feeding ancestor of all modern psyllids; however, they also mentioned in the last sentence of that paragraph that “*Togepsylla* (Aphalaridae), which feeds on Lauraceae (Annonales), may possibly be a relic genus of a psyllid group which antedates the Rutales-feeders.” On the other hand, using the now synonymized *Hemipteripsylla* as the type genus, Li (2011) elevated Togepsyllinae to superfamily level, Hemipteripsylloidea, containing a single family with three genera. He recognized Hemipteripsylloidea as a primitive member of ‘Psyllidomorpha’ without a supporting phylogenetic analysis, simply based on the flat-held wings and one-segmented instead of two-segmented aedeagus in males. In the most recent, influential classification of Burckhardt and Ouvrard (2012), Togepsyllinae was treated as one of the five subfamilies of Aphalaridae whose fifth-instar immatures lack an unguitractor on the tarsal arolium, closer related with Rhinocolinae and Spondyliaspidinae.

Based on a synthesis of fossil studies, the defining character of Psylloidea *sensu stricto* is the mutual modification of the metacoxa and furca of the metathorax (Ouvrard et al. 2010) to allow the attachment of jumping muscles (Bekker-Migdisova 1971), thus enabling psyllids to jump. According to limited observations, psyllids appears to do a mid-air somersault in jumping. The jumping of *Psylla alni* (Linnaeus), *Cacopsylla peregrina* (Foerster) and *Psyllopsis fraxini* (Linnaeus) is triggered by pressing hind trochanters and femora against the coxal wall, then a quick release of the tension, powered by the metathoracic internal muscles attached to the furca and trochanteral tendon (Burrows 2012). The release is strong enough to rotate the entire body upside down, but the fore legs provide a support to maintain balance, and then the entire insect is cast airborne and starts to rotate forward rapidly, sometimes with the wings opening in mid-air to commence flight. The “non-jumping plant lice” of the extinct families Liadopsyllidae and Malmopsyllidae, which date back to the Mesozoic and possess unmodified metacoxa, provides further support that the modified hind legs are monopolized by modern Psylloidea (Ouvrard et al. 2010). Occasionally, some extant psyllids, such as Togepsyllinae and *Apsylla cistellata* (Buckton 1896) (Aphalaridae: Rhinocolinae), are not known to jump. These exceptions are regarded by Ouvrard et al. (2010) as secondary reductions.

A new species, *Togepsylla glutinosae* sp. n., has been collected on *Litsea glutinosa* from tropical China recently. During the taxonomic work on Chinese Aphalaridae, many previously overlooked characters of *Togepsylla* were revealed, including the half-modified metacoxae which do not provide sufficient jumping force, and wax secreting fields on abdominal sternites first found on a psyllid. Based on an integration of East Asian collections, this study aims to revise the taxonomy of the genus *Togepsylla*, and reassess the distinctive morphological characters of it.

## Materials and methods

This study is based on the collections of the Entomological Museum of China Agricultural University (**CAU**), the Natural History Museum, London (**BNHM**), Hiromitsu Inoue’s personal collection (**HIC**), and the Osaka Museum of Natural History (**OMNH**). The type series of *Togepsylla takahashii* and *T. matsumurana*, previously preserved in Hokkaido University, were lost according to Jin Hyung Kwon (personal communication). Specimens examined regarding the non-*Togepsylla* species involved in discussion are listed as follows:

**BNHM** *Atmetocranium myersi* Ferris & Klyver, 1932: 1 ♂;

**CAU** *Cacopsylla* sp.: numerous adults of both sexes;

**BNHM** *Syncoptozus mexicanus* Hodkinson, 1990: 1 ♂;

**CAU** *Trialeurodes vaporariorum* Westwood, 1856: numerous adults of both sexes.

Slides were prepared following this protocol: whole insect soaked in boiling potassium hydroxide (KOH) solution for 10 minutes, naturally cooled down after heating stopped, washed in distilled water, and finally mounted on a slide in glycerine. All drawings and examinations were undertaken with an Olympus BX41 microscope. One dry-mounted adult of each *Togepsylla* species was coated with gold using a Leica EM SCD050 Super Cool Sputter Coater and prepared for Scanning Electron Microscope (SEM) examination and photography, using an FEI^®^ Quanta 450 Environmental Scanning Electron Microscope.

Measurements were taken with a Keyence VHX-1000 digital microscope using the measuring function and are given in millimeters (mm). For adults:

**HW** head width,

**AL** antennal length,

**SL** length of the posterior pair of prickly setae on the median of the vertex,

**TW** mesoscutum width,

**WL** fore wing length,

**TL** metatibial length.

For fifth instar immatures:

**BL** total body length,

**AL** antennal length,

**HW** head width,

**FL** fore wing pad length, measured as the distance between the transverse tangents of the anterior angle and posterior margin.

Total body length of adults was not determined because most dry-mounted specimens examined were in distinct positions and therefore incomparable.

Comparisons of morphological characters were based on direct observations of specimens, drawings, SEM photos, measurements, and sometimes on the literature. Putatively homologous characters were compared across the concerned taxa. Diagnostic characters are described for each species, with emphasis on the differences. Characters potentially useful for systematic studies are noted and are referred to in the discussion.

Terminology primarily follows Brown and Hodkinson (1988), Ouvrard et al. (2002), and Yang et al. (2009) for adults and White and Hodkinson (1985) for immatures.

Scientific names of plants follow the Missouri Botanical Garden (2016), and higher systematics of plants (except for the direct citation of older literatures) follow The Angiosperm Phylogeny Group (2016).

## Taxonomy

### Togepsylla

Taxon classificationAnimaliaHemipteraPsylloidea

Kuwayama, 1931

Togepsylla Kuwayama, 1931: 121. Type species: Togepsylla takahashii Kuwayama, 1931, by original designation.
Togepsylla Kuwayama: Li 2011: 213.
Hemipteripsylla Yang & Li, 1981: 182. Type species: Hemipteripsylla tibetana Yang & Li, 1981, by original designation. Synonymized by Hodkinson 1990: 716.

#### Diagnosis.

Body relatively flat. Vertex, thoracic dorsum and most of fore wing veins with symmetrical long and thick setae, which possess tiny spinules on the surface (Fig. 63, termed ‘prickly setae’ below). Wings held flat over back. Fore wing lacking pterostigma. Hind wing with a single thick anal vein (A) which may result from the reduction of vein A_1_ or A_2_ or from the combination of them. Posterior aspect of male proctiger enveloped. Aedeagus uni-segmented. Female subgenital plate simple and situated much more proximal than proctiger. Valvula dorsalis of ovipositor lacking flag lobe. Fifth instar immature with symmetrical sectasetae on body dorsum, lacking tarsal arolium on legs.

#### Redescription.

***Adult.*** Body flat, with abdomen significantly wider than tall. Body dorsum with symmetrical prickly setae on the surface, situated on bulges or projections, distribution as: 4+4 on vertex, 4+4 on pronotum, 1+1 on mesopraescutum, 4+4 (Fig. 53) or 5+5 (Fig. 52) on mesoscutum, 1+1 on mesoscutellum, 1+1 on tegula, 1+1 on humeral plates, 1+1 on metascutellum. Surface of vertex and thoracic dorsum sculptured with granular microscopic structures.

Head slightly inclined from longitudinal body axis. Vertex lacking median suture; two tubercles present along the median line, each bearing a pair of prickly setae. Base of lateral ocelli moderately bulging, each bearing two prickly setae. Vertex consistent with gena. Plane of torulus about perpendicular to that of the vertex. Frons completely fused with vertex and gena, only moderately raised from the surface. Gena not divided into two lateral parts, but firmly compact as one, with roughly symmetrical simple setae (Fig. 47); parts below torulus sometimes produced. Occiput smoothly connected with vertex, not folded below it. Plane of postocular sclerite about perpendicular to that of vertex, not nearly parallel with it. Antennae 10-segmented, surface sculptured with minute spinules arranged in transverse rows; at least six rhinaria present on apices of segments IV-IX, apex of segment III sometimes also with one; segments IV, VI and VIII sometimes possess extra rhinaria; rhinarium with closely packed minute spinules lining below, usually bearing horn-like projections. Clypeus rather short, with no extra seta except the apical pair of setae. Labium rather short, two-segmented, lacking ‘conical sensoria’ (as termed by Liang et al. 2013) on the tip (Fig. 48).

Preepimeron significantly wider than preepisternum. Notopleural sulcus of prothorax well developed. Mesopraescutum near semicircular, not protruding forward to force pronotum to arch. Pleural sulcus of mesothorax reduced, with pleural apophysis relatively small; posterior margin of mesopleurite directed forward. Mesepisternum rather narrow and bulging (Fig. 57). Trochantinal apodeme shallow, present on anterior margin of mesopleurite (Fig. 57). Anapleural cleft of mesothorax widely split (Fig. 57). 1+1 extra sclerites present behind the base of mesothoracic furca (Fig. 59). Heel of mesepimeron swollen, bearing a small tubercle (Fig. 57). Metathoracic pleural sulcus reduced, pleural apophysis poorly developed; metepisternum and trochantin not completely divided (Fig. 57). Trochantinal apodeme of metathorax shallow, present on the anterior margin of metapleurite (Fig. 57). Katepisternum and trochantin of metathorax possess well developed ventral aspect which are convergent in the middle, forming a large and solid plate ventrally (Fig. 59). 1+1 extra sclerites present behind the base of metathoracic furca (Fig. 59) (in contrast with most other psyllids, e.g. *Cacopsylla*, Fig. 60).

Legs long and slender. The three sensory pores on femora ventrum arranged in a row. Plane of hind legs almost parallel with that of middle legs. Metacoxa with rather large tubercle above apical opening, and lacking meracanthus (Fig. 56). Metafemur without a cluster of thick setae on the outside of apex. Metatibia without genual spine, with 1-3 rows of thick setae; apical part often with a row of tightly packed setae dorsally; apical spurs relatively long and slender, sclerotized at different extents but never reaching the hard and black status as in most other psyllids, forming an open crown. Metabasitarsus lacking sclerotized spurs on the apex, but with one or two (in other psyllids there is only one) pairs of simple setae. Apical tarsus with a pair of short and tapered apical setae. Claws with rounded or rather narrow pulvilli.

Fore wing narrowest in the base and gradually becoming much wider apically, usually widest at subapex or apical 1/4. Costal break present. Pterostigma absent. Vein Rs reaching anterior margin instead of apical margin. Cell cu_1_ rather long and flat. Veins A_1_ and A_2_ touching in the middle. Anal break adjacent to the apex of vein Cu_1b._

Hind wing with partially thickened anterior margin. Veins A_1_ and A_2_ combined or one is lost (probably A_1_), leaving a thickened vein A; cell a_1_ lost (Fig. 49).

Tergite of abdominal segment 1 better developed, with a median sclerite present (Fig. 50). Spiracles of segments 1 and 2 invisible. Sternites of segments 4-6 each with a pair of wax-secreting pore fields laterally, with shape variable.

Male terminalia: In natural status, proctiger, aedeagus and parameres all oriented caudally instead of upwards. Posterior aspect of proctiger enveloped. Aedeagus uni-segmented and simple, sometimes with tiny spines on dorsum. Sperm pump with only basal end plate, lacking apical end plate (Fig. 51).

Female terminalia: Subgenital plate placed much more proximal than proctiger and simple, lacking tip sometimes. Proctiger lacking rows of long setae on the dorsum. Valvula dorsalis of ovipositor without flag lobe. Median valve slender and placed more terminal, apex touching the subapex of ovipositor.

***Fifth instar immature.*** Body dorsum with symmetrical sectasetae. Antennal 7- or 9-segmented, with three rhinaria. Compound eyes with 1+1 or 2+2 ocular setae. Postocular setae present in 2+2 or more. Fore wing pads simple, without humeral lobe. Legs long and slender, lacking specialized seta. Both tarsal segments differentiated. Tarsal claws with pulvilli and without arolium. Apical setae of tarsus both long and capitate. Abdominal sclerites firm, not broken in the middle. Abdominal apex with a pair of bulges. Circum anal pore field lacking additional rings.

#### Key to adults of *Togepsylla*

**Table:** 

1	Mesoscutum with 5+5 prickly setae (Fig. 52). Antennal segment III lacking rhinarium on the apex	**2**
–	Mesoscutum with 4+4 prickly setae (Fig. 53). Antennal segment III with one rhinarium on the apex	**3**
2	Fore wing colorless, with one prickly seta on the base of vein M_3+4_ (Fig. 12). Inner surface of paramere with large area of netlike grains covering the whole apical half, anterior margin serrated (Fig. 28). Female terminalia rather small compared with body size, proctiger curved upwards only at the tip (Fig. 32)	***Togepsylla tibetana* (Yang & Li)**
–	Fore wing with black sections on veins, without prickly setae on vein M_3+4_ (Fig. 10). Inner surface of paramere with a small area of netlike grains in the centre, anterior margin not serrated (Fig. 23). Female terminalia relatively large compared with body size, apical 1/3 of proctiger strongly curved upwards (Fig. 30)	***Togepsylla matsumurana* Kuwayama**
3	Fore wing with yellow bands, with rather long prickly setae on veins but M_3+4_ (Fig. 11). Dorsum of metatibia with a closely packed row of short setae (Fig. 15). Paramere with a sclerotized tooth anteriorly (Figs 25, 26). Female proctiger smoothly tapered apically (Fig. 31)	***Togepsylla takahashii* Kuwayama**
–	Fore wing without color patterns, with relatively short prickly setae on veins including M_3+4_ (Fig. 9). Dorsum of metatibia lacking a closely packed row of short setae (Fig. 13). Paramere without sclerotized tooth (Figs 19–21). Female proctiger constricted at apical 1/3 (Fig. 29)	***Togepsylla glutinosae* sp. n.**

#### Key to the fifth instar immature of *Togepsylla* (*T. tibetana* unknown)

**Table:** 

1	Body dorsum with acute-tipped sectasetae (Figs 38, 40). Circum anal ring strongly winding, expanded in lateral aspect (Fig. 42)	***Togepsylla takahashii* Kuwayama**
–	Body dorsum with truncate sectasetae. Circum anal ring simple, both outer and inner rings composed of single row of pores.	**2**
2	Outer margin of head and fore wing pad with closely packed sectasetae (Fig. 43)	***Togepsylla matsumurana* Kuwayama**
–	Outer margin of head and fore wing pad with fewer and scattered sectasetae (Figs 33, 35)	***Togepsylla glutinosae* sp. n.**

### Togepsylla glutinosae sp. n.

Taxon classificationAnimaliaHemipteraPsylloidea

http://zoobank.org/F4C2FD9E-E5BC-4F35-B7AA-AA10A2F69194

Figs 1 , 5 , 9 , 13 , 16 , 19–21 , 29 , 33–37 , 64 , 65

#### Diagnosis.

Vein M_3+4_ of fore wing with 3 prickly setae (Fig. 9). Tarsal pulvilli rounded (Fig. 13). Male subgenital plate without long seta on the dorsal-apical angle (Fig. 19). Female proctiger steeply narrowed in the apical 1/3 (Fig. 29).

#### Description.

***Adult coloration.*** Ground color yellow. Long and thick setae on dorsum black. Compound eyes grey. Ocelli yellow. Antennae yellow, with black apices on segments IV, VI, VIII; segments IX-X entirely black. Pronotum, meso- and metascutum each with one pair of orange markings. Legs yellow. Fore wing hyaline and colorless (Fig. 9). Tergites of abdominal segments 3-5 brown. Male and female terminalia yellow.

**Figure 1–8.**
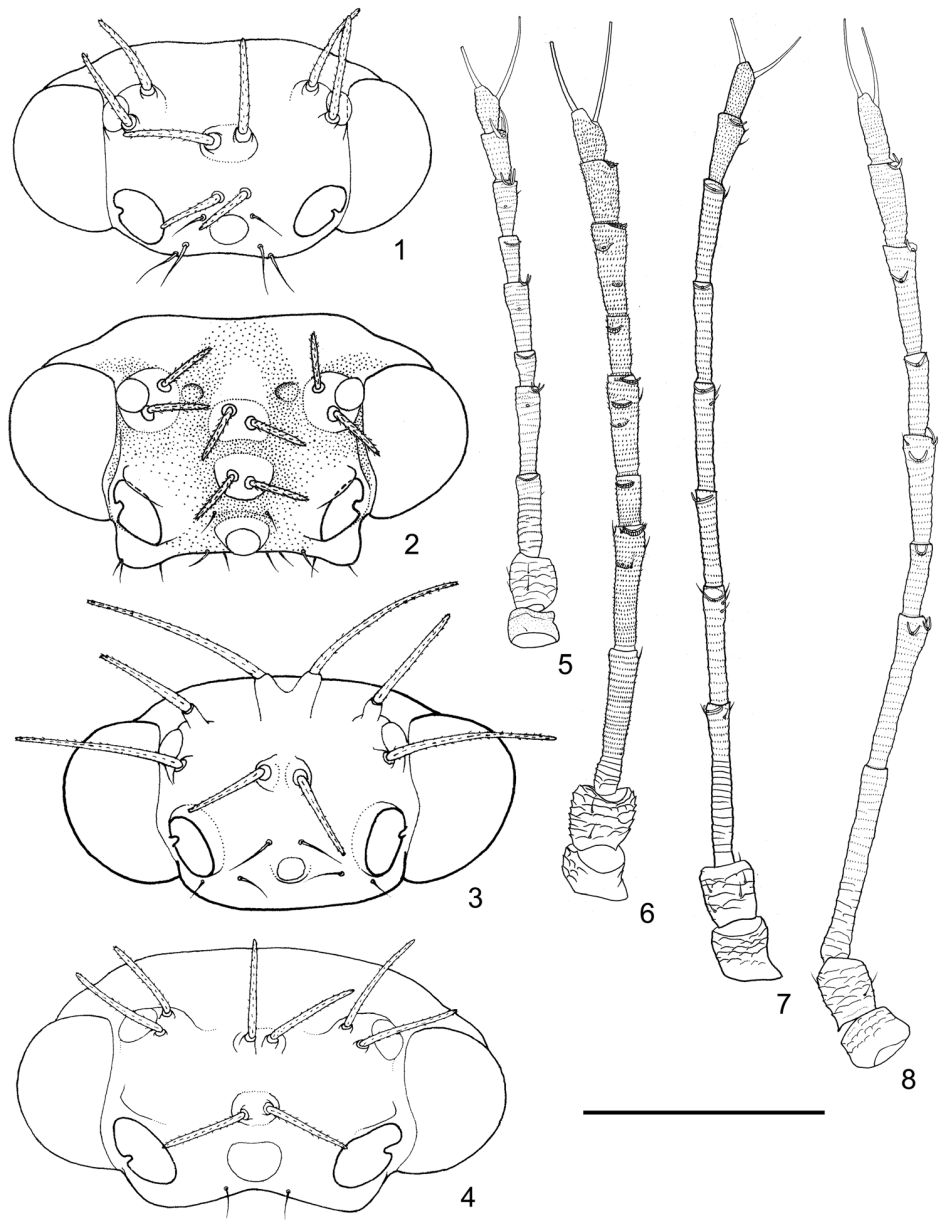
Head and antennae of *Togepsylla* spp. **1, 5** *Togepsylla glutinosae* sp. n. **2, 6** *Togepsylla matsumurana* **3, 7** *Togepsylla takahashii* **4, 8***Togepsylla tibetana* **1–4** Head **5–8** Antenna. Scale bar: 0.2 mm.

Structures: Setae on dorsum of body relatively long (Table 1) and based on prominent projections. Gena flat (Fig. 1). Antennal segments III-IX each with a single rhinarium on apex, segments IV, VI and VIII each with one extra rhinarium; rhinarium on segment IX double-pored and with complex horn-shaped projections; proximally based terminal seta about twice as long as the distally based one (Fig. 5).

**Table 1.** Measurements in mm.

**Adults**		**HW**	**AL**		**SL**	**TW**	**WL**		**TL**
*Togepsylla glutinosae*	4♂♂	0.34–0.36	0.36–0.47		0.65–0.92	0.32–0.35	1.00–1.08		0.40–0.45
	2♀♀	0.36–0.38	0.40–0.48		1.04–1.06	0.38–0.39	1.23–1.24		0.46–0.48
*Togepsylla matsumurana*	4♂♂	0.42–0.44	0.71–0.76		0.80–0.88	0.49–0.50	1.84–1.92		0.70–0.72
	4♀♀	0.44–0.46	0.65–0.73		0.83–0.95	0.52–0.56	2.10–2.23		0.59–0.67
*Togepsylla takahashii*	4♂♂	0.36–0.38	0.87–0.92		1.37–1.54	0.36–0.38	1.38–1.41		0.56–0.59
	3♀♀	0.39–0.41	0.92–0.96		1.47–1.58	0.42–0.44	1.53–1.62		0.67–0.69
*Togepsylla tibetana*	4♂♂	0.42–0.43	0.77–0.80		0.74–0.81	0.40–0.48	1.80–2.01		0.64–0.72
	4♀♀	0.42–0.44	0.71–0.85		0.83–0.88	0.45–0.48	1.98–2.20		0.60–0.64
**Fifth instar immatures**		**BL**		**AL**		**HW**		**FL**	
*Togepsylla glutinosae*	n = 5	0.89–0.99		0.34–0.38		0.29–0.33		0.30–0.35	
*Togepsylla matsumurana*	n = 2	1.14–1.30		0.38–0.47		0.35–0.42		0.42–0.51	
*Togepsylla takahashii*	n = 5	1.32–1.46		0.56–0.61		0.40–0.41		0.46–0.52	

**Figure 9–12.**
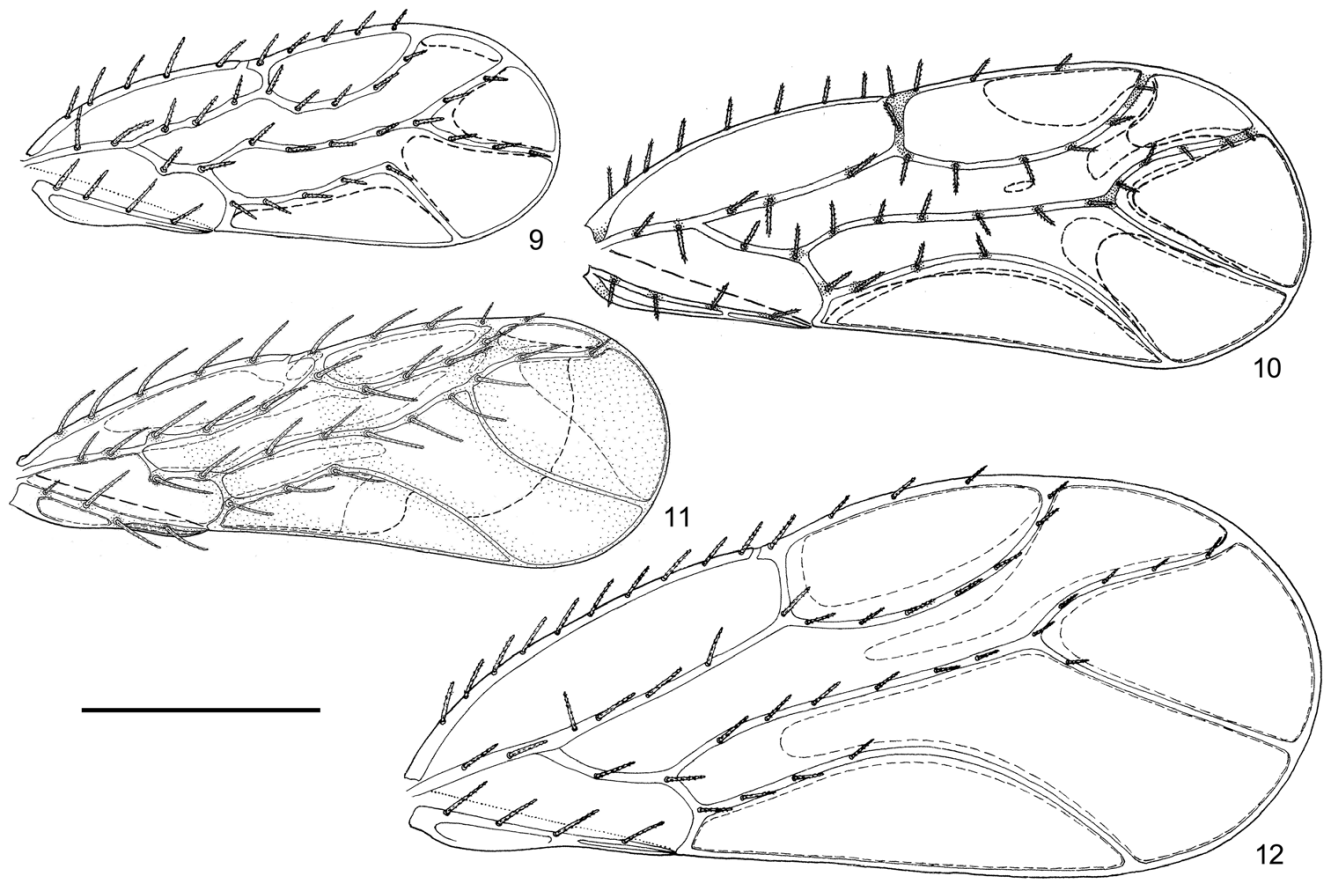
Fore wing of *Togepsylla* spp. **9** *Togepsylla glutinosae* sp. n. **10** *Togepsylla matsumurana* **11** *Togepsylla takahashii* **12** *Togepsylla tibetana*. Scale bar: 0.5 mm.

**Figure 13–18.**
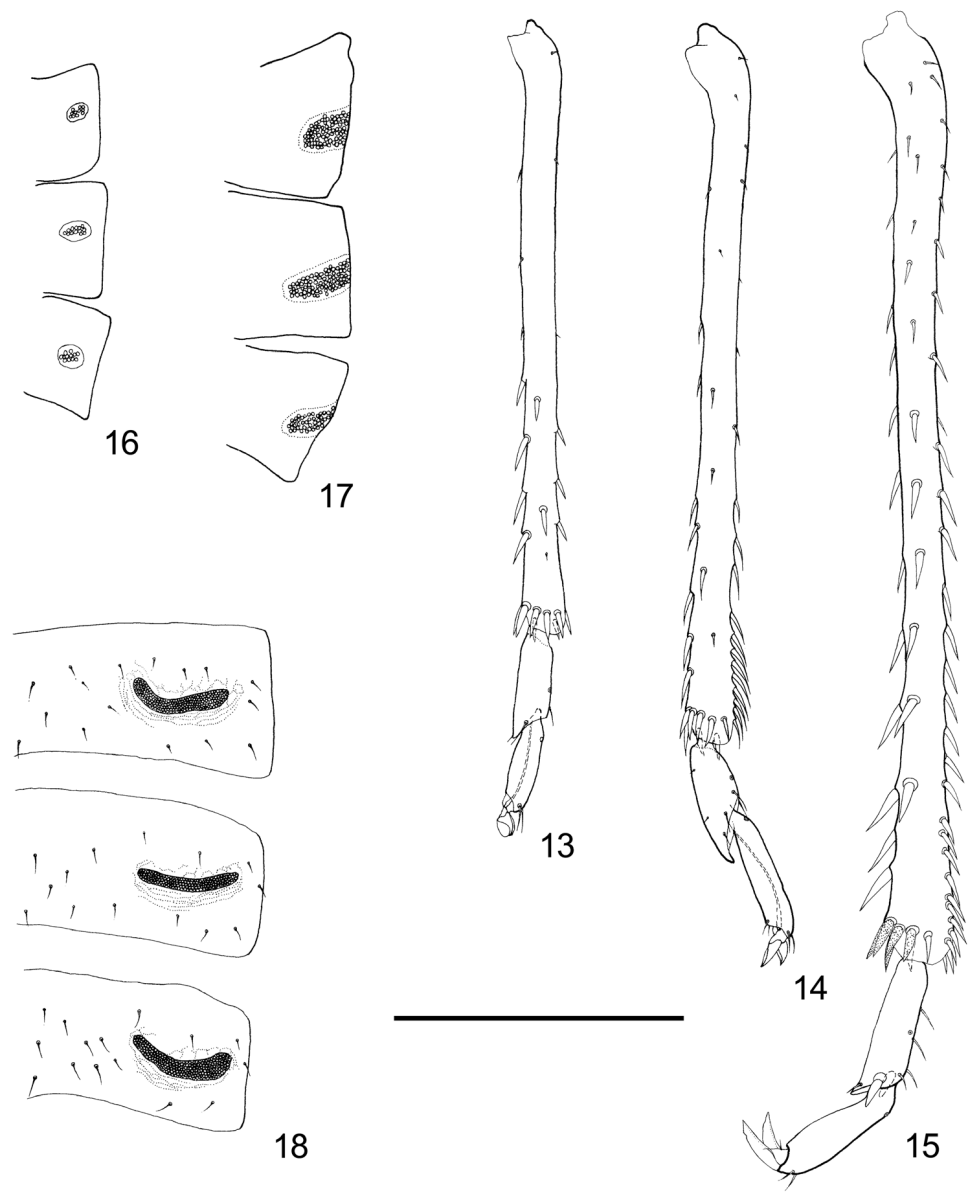
Hind legs and wax-secreting fields of *Togepsylla* spp. **13, 16** *Togepsylla glutinosae* sp. n.; **14, 17** *Togepsylla matsumurana* **15, 18** *Togepsylla takahashii* **13–15** Metafemora and tibia **16–18** Wax-secreting fields on lateral aspect of sternites of abdominal segments IV–VI. Scale bar: 0.2 mm.

Mesoscutum with four pairs of prickly setae. Metatibia with three short rows of thick setae, lacking a tightly packed row of short setae on the dorsum (Fig. 13). Apex of metabasitarsus with only one pair of simple setae (Fig. 13). Pulvilli broadly rounded (Fig. 13). Fore wing cell cu_1_ tallest in apical 1/3, with vein Cu_1a_ abruptly curved at the point; vein M_3+4_ completely decorated with setae; surface spinules absent; fields of radular spinules relatively large (Fig. 9).

Pore fields on abdominal ventrum small oval; pores loosely packed (Fig. 16).

**Figure 19–28.**
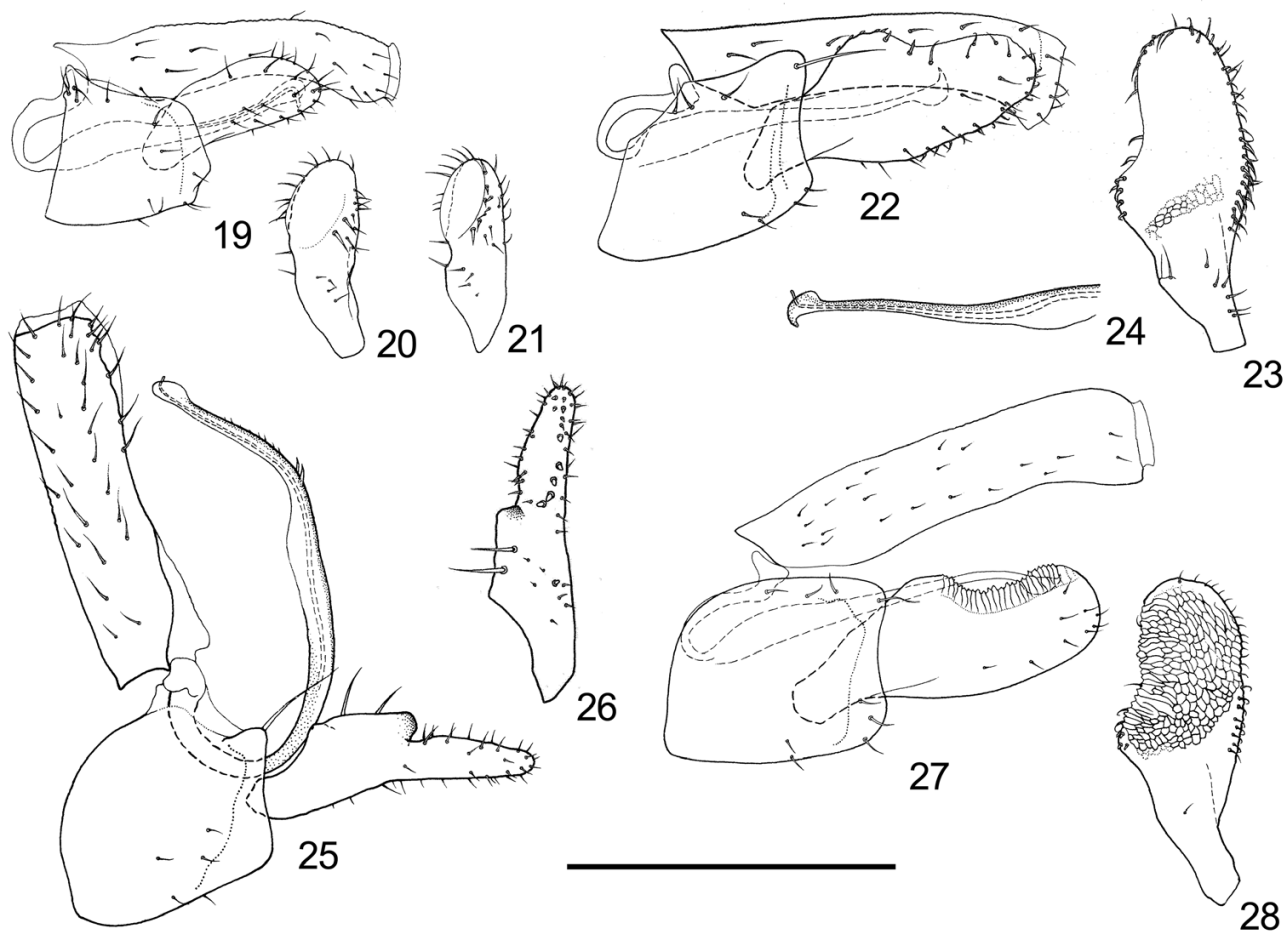
Male terminalia of *Togepsylla* spp. **19–21** *Togepsylla glutinosae* sp. n. **22–24** *Togepsylla matsumurana* **25, 26** *Togepsylla takahashii* **27, 28** *Togepsylla tibetana* **19, 22, 25, 27** Male terminalia, in profile **20, 23, 26, 28** Paramere, inner surface **21** Paramere, posterior view **24**. Apical half of aedeagus. Scale bar: 0.2 mm.

Male terminalia: Proctiger slightly curved backwards (Fig. 19). Paramere small lamellar; apical half of anterior margin with a thin lobe stretching inwards; anterior margin of basal 1/3 emarginated; two long and thick setae present on inner surface, near the anterior margin; inner surface with a curved ridge decorated with thick setae on apical half (Figs 19–21). Aedeagus with a few tiny spines on the dorsum (Fig. 19). Base of subgenital plate with a small cluster of setae (Fig. 19).

**Figure 29–32.**
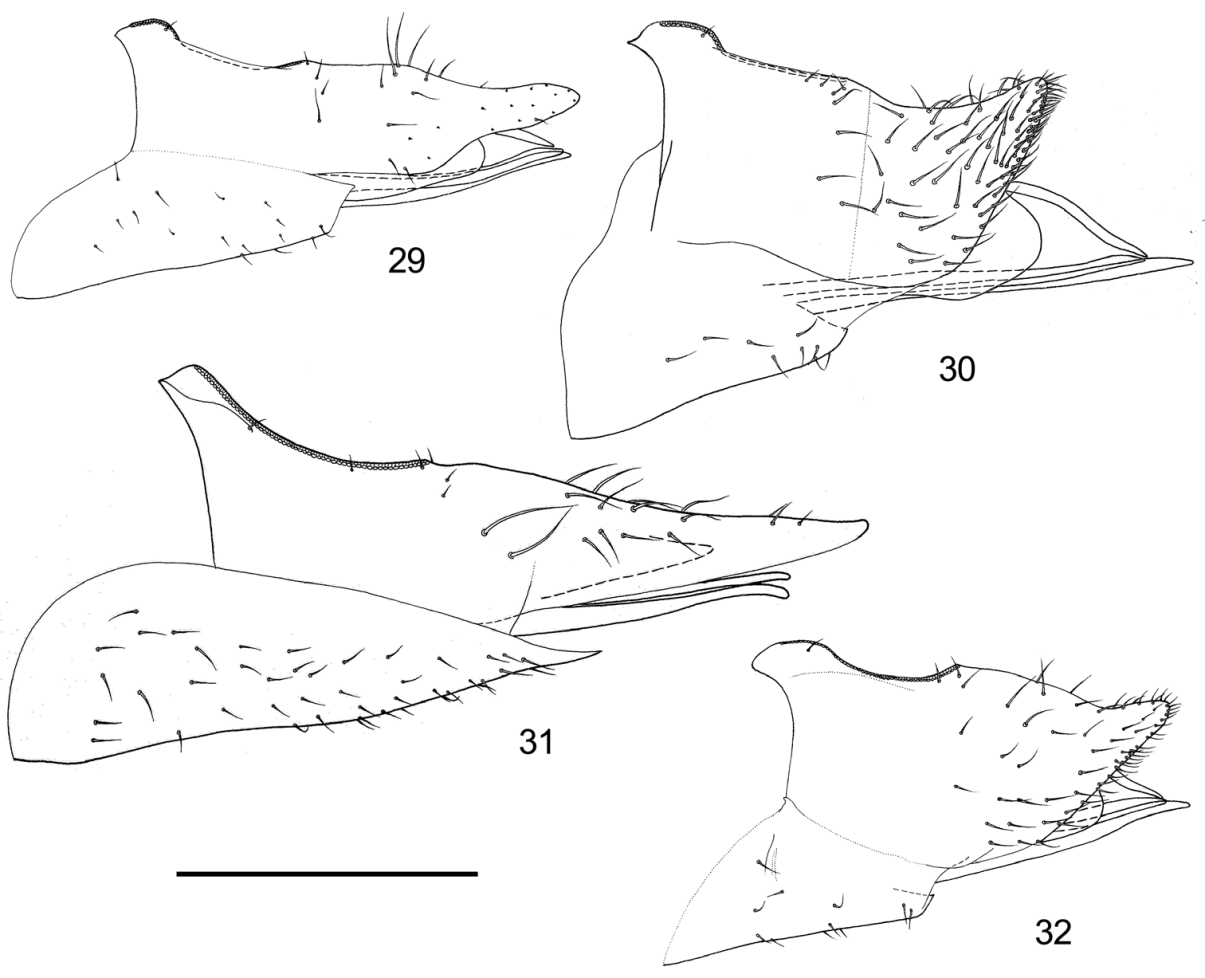
Female terminalia of *Togepsylla* spp. **A** *Togepsylla glutinosae* sp. n. **B** *Togepsylla matsumurana* **C** *Togepsylla takahashii* **D** *Togepsylla tibetana*. Scale bar: 0.2 mm.

Female terminalia (Fig. 29): Oblong in overall shape. Base of proctiger slight raised, anus partly sunken; dorsal view of proctiger constricted at apical 1/3; apical process with small amounts of tiny setae. Subgenital plate lacking tip, with sparse setae on ventral surface.

**Figure 33–42.**
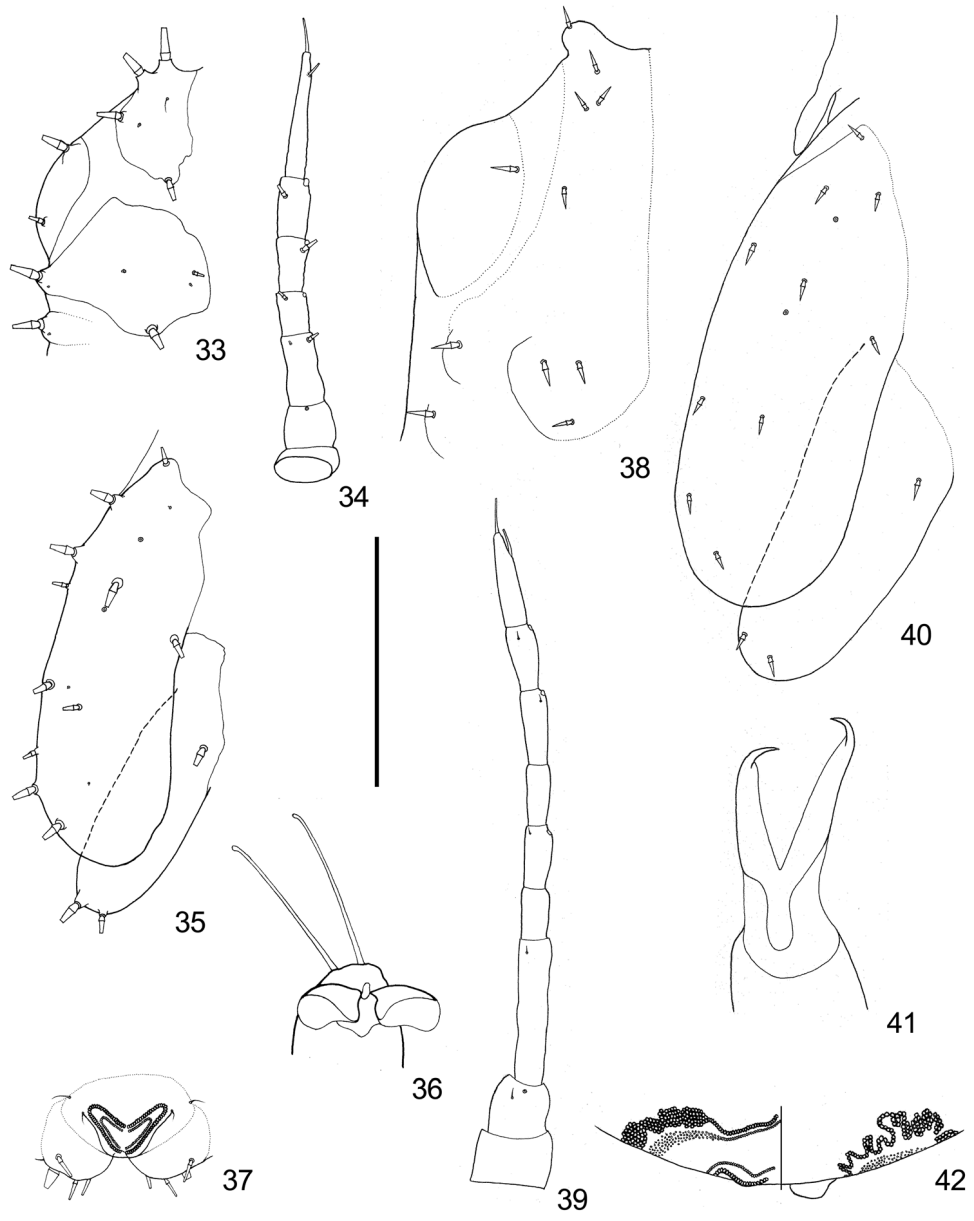
Fifth instar immature of *Togepsylla* spp. **33–37** *Togepsylla glutinosae* sp. n. **38–42** *Togepsylla takahashii* **33, 38** Half of head, dorsal view **34, 39** Antenna, dorsal view **35, 40** Wing pads, dorsal view **36** Claws, showing pulvilli and apical setae of tarsus **37** Circum anal ring, ventral view **41** Claws, showing pulvilli **42** Circum anal ring, dorsal view on the left half, ventral view on the right half. Scale bar: 0.2 mm (**33–35, 37, 38–40, 42**), 0.05 mm (**36, 41**).

***Fifth instar immature.*** Body dorsum strongly sclerotized, ventrum weakly sclerotized. Dorsum of head, thorax and abdomen with symmetrical truncate sectasetae varying in size, mixed with a few simple setae (Fig. 33); dorsum and margin of wing pads with roughly symmetrical truncate sectasetae (Fig. 35). Antennae 7-segmented, apices of segments 4-6 each with one single rhinarium, segments 3-6 each with one single truncate sectaseta (Fig. 34). Compound eyes with 2+2 ocular truncate sectasetae, postocular truncate sectasetae present in 2+2 (Fig. 33). Fore wing pad with two pores on dorsum (Fig. 35). Tarsal pulvilli broad and rounded (Fig. 36). Abdominal ventrum with four pairs of spiracles surrounded by peritremes partly fused with central sclerites. Abdominal apex produced as a pair of rounded bulges (Fig. 37). Circum anal pore field present in between the bulges, both outer and inner ring consisting of neat single row of oval pores (Fig. 37).

#### Material examined.

Holotype: ♂, CHINA: Hainan, Danzhou, Nada, 131 m, 19°30.878'N, 109°31.085'E, ex *Litsea glutinosa*, 12.iv.2016, Xinyu Luo (CAU). Paratypes: 10 ♂, 12 ♀, 15 immatures, same data as holotype (CAU).

#### Host plant.

*Litsea glutinosa* (Lour.) C. B. Rob. (Lauraceae)

#### Distribution.

**China**: Hainan.

#### Etymology.

Named after the scientific name of the host plant.

#### Biology.

Based on a brief observation in the field, this species was found free living, both immatures and adults are sparsely scattered across the abaxial surface of leaves (no preference for young leaves or shoots is displayed). The immatures do not induce any form of gall or leaf rolling, and from the sectasetae on body margin they produce wax threads of varying lengths, of which the ones from the terminal bulges of abdomen are longest (Fig. 65).

### Togepsylla matsumurana

Taxon classificationAnimaliaHemipteraPsylloidea

Kuwayama, 1949

Figs 2 , 6 , 10 , 14 , 17 , 22–24 , 30 , 43–46 , 52 , 62–63

Togepsylla matsumurana Kuwayama, 1949: 48; Miyatake 1970: 1; Yang 1984: 192.
Togepsylla matsumurai Kuwayama: Miyatake 1981: 52. Misspelling.
Hemipteripsylla matsumurana (Kuwayama): Yang and Li 1981: 182; Li 2011: 212.
Togepsylla zheana Yang, 1995: 109. Synonymized by Li 2011: 212.

#### Diagnosis.

Dorsum of head and thorax brown with large areas of brown patterns. Antennal segments VI and VIII each with two additional rhinaria (Fig. 6). Paramere with a small area of netlike grains on inner surface (Fig. 23). Apical 1/3 of female proctiger strongly curved upwards (Fig. 30).

#### Redescription.

***Adult coloration.*** Head yellow, vertex with brown patterns. Long and thick setae on dorsum black. Compound eyes light brown. Ocelli yellow. Antennae yellow, segments I-II light brown, apices of segments III, IV, VI, VIII black, segments IX-X entirely black. Thoracic dorsum brown, except for bases of setae which are yellow. Thoracic pleurites light brown. Legs yellow, with apical half of femora light brown, apex of tibiae brown. Fore wing membrane hyaline and colorless; R_1_, apices of Rs and M_1+2_ black (Fig. 10). Abdominal tergites of segments 1-5 black, sternites brown. Male proctiger brown. Female terminalia yellow.

Structures: Setae on dorsum of body relatively short (Table 1) and based on smooth projections. Vertex with a pair of small foveae between median-posterior tubercle and lateral ocelli (Fig. 2). A pair of small tubercles present above toruli (Fig. 2). Genal tubercles strongly protruding (Fig. 2). Antennal segments IV-IX each with a single rhinarium on apex, segment IV with one, segments VI and VIII each with two extra rhinaria; rhinaria without horn-shaped projection; proximally based terminal seta slightly longer than the distally based one (Fig. 6).

Mesoscutum with 5 pairs of prickly setae (Fig. 52). Metatibia with one row of thick setae ventrally, and with a tightly packed row of long setae on the dorsum (Fig. 14). Pulvilli narrow (Fig. 14). Fore wing with broad cell r_1_, cell cu_1_ tallest in the middle; vein M_3+4_ without seta; surface spinules rather minute, widely spread across a relatively small area in distal cells; fields of radular spinules relatively large (Fig. 10).

Pore fields on abdominal ventrum large oval, with pores loosely packed (Fig. 17).

Male terminalia: Proctiger slightly curved backwards apically (Fig. 22). Paramere broad lamellar, with rather slender base; anterior margin of apical half emarginated and thin; posterior margin with a basal ridge on outer surface; middle of inner surface with a small area of netlike grains; anterior angle with a few short and thick setae on inner surface; posterior margin with a band of inner-curved short setae on apical 2/3 (Figs 22, 23). Tip of aedeagus forming an acute small hook, dorsum of aedeagus lacking tiny spines (Fig. 24). Subgenital plate with moderately produced dorsal-apical angle, and with a few setae on the base (Fig. 22).

Female terminalia (Fig. 30): Short and broad in overall shape. Apical 1/3 of proctiger strongly curved upwards; apical half of proctiger with nearly evenly spaced setae, and with a row of setae along ventral margin of apical process. Subgenital plate with blunt and retracted apex, ventral surface with sparse setae.

**Figure 43–46.**
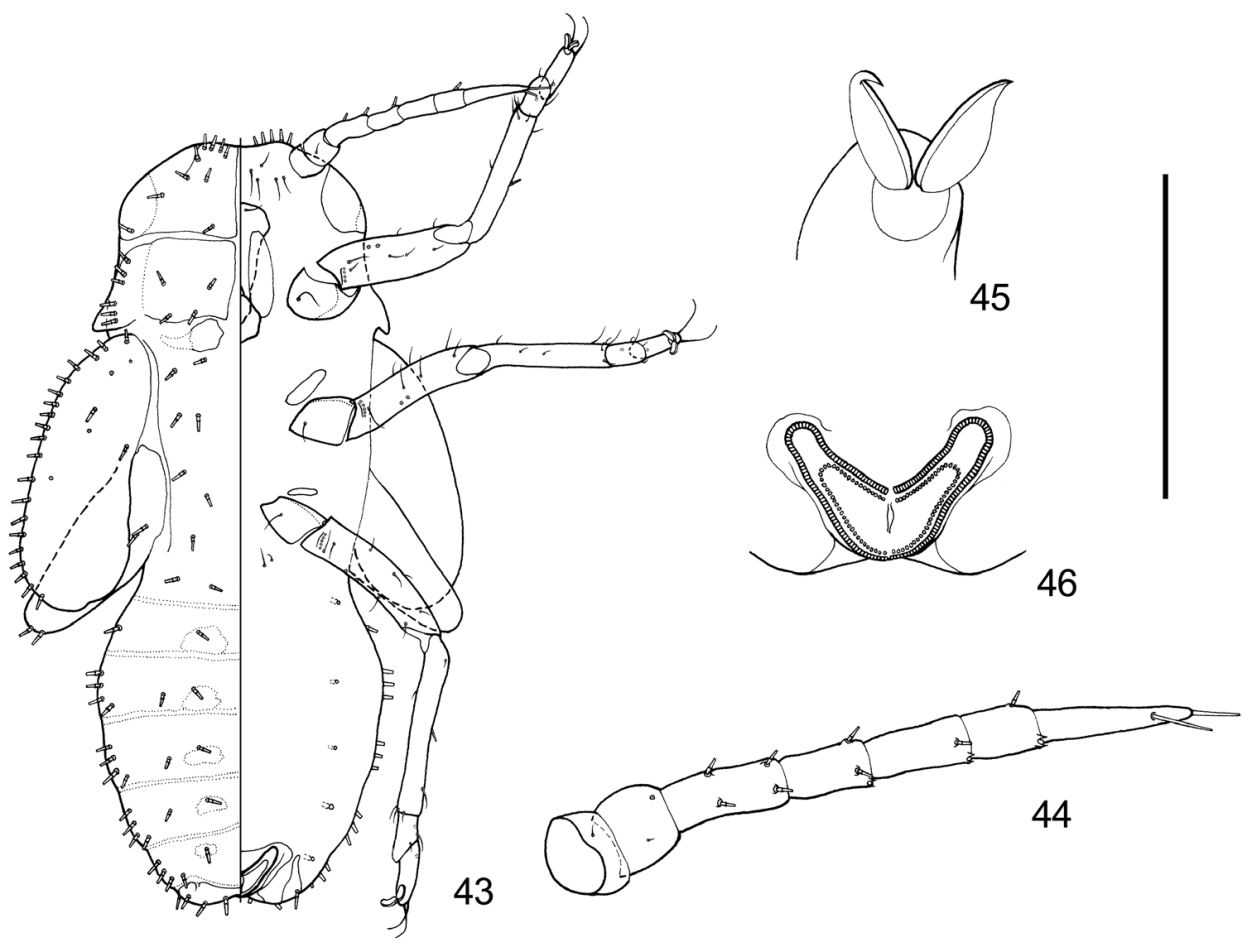
Fifth instar immature of *Togepsylla matsumurana*. **43** Overall view, dorsal view on the left half, ventral view on the right half **44** Antenna, dorsal view **45** Claws, showing pulvilli **46** Circum anal ring, ventral view. Scale bar: 0.5 mm (**43**), 0.2 mm (**44, 46**), 0.05 mm (**45**).

***Fifth instar immature.*** Body dorsum firmly sclerotized, with sclerites of thorax and abdomen almost unseparated; body ventrum weakly sclerotized (Fig. 43). Dorsum of head, thorax, and abdomen with symmetrical truncate sectasetae varying in size (Fig. 43). 1+1 projections present before fore wing pads, sheathing the 2+2 long setae on lateral margins of adult pronotum (Fig. 43). Antennae 7-segmented, apices of segments 4-6 each with one single rhinarium; segment 3-6 with truncate sectasetae on dorsum (Fig. 44). Compound eyes with 1+1 ocular truncate sectasetae, postocular truncate sectasetae present in 6+6 (Fig. 43). Fore wing pad with three pores on dorsum, and with outer margin completely decorated with truncate sectasetae (Fig. 43). Tarsal pulvilli narrow (Fig. 45). Abdominal ventrum with five pairs of spiracles (Fig. 43). Abdominal apex emarginated (Fig. 46). Circum anal pore field with both outer and inner ring consisting of neat single row of oval pores (Fig. 46).

#### Material examined.

CHINA: 2 ♀, Zhejiang, Qingyuan, Baishanzu, 1300-1500 m, ex *Litsea cubeba*, 24.ix.1993, Hong Wu (CAU) (type series of *Togepsylla zheana*); 1 ♂, 2 ♀, Yunnan, Yiliang, Xiaobanchang, 1883 m, 27°48.227'N, 104°21.155'E, 27.iv.2014, Xinyu Luo (CAU); 2 ♂, 3 ♀, Guangxi, Wuming, Mt. Daming, 1341 m, 23°30.421'N, 108°26.084'E, 12.v.2014.v.12, Xinyu Luo (CAU). JAPAN: 2 ♂, 2 ♀, 5 fifth instar immatures, Ibaraki Prefecture, Tsuchiura City, Shishisuka, 15 m, 36°4.8'N, 140°9.54'E, ex *Neolitsea sericea*, 29.iv.2004, Hiromitsu Inoue (HIC); 3 ♂, 3 ♀, Tochigi Prefecture, Kaminokawa, Kamigo, 65 m, 36°26.4'N, 139°55.98'E, ex *Neolitsea sericea*, 24.iii.2015, Rikio Sonobe (HIC); 1 ♂, 1 ♀, Fukuoka Prefecture, Mt. Hiko, 26.iv.2001, Hiromitsu Inoue (HIC).

#### Host plant.

*Litsea cubeba* (Lour.) Pers. (Yang 1995), *Lindera erythrocarpa* Makino, *Lindera glauca* (Zieb. et Zucc.) Bl (Miyatake 1970), *Lindera* sp. (Miyatake 1981), *Neolitsea sericea* (Bl.) Koidz (HIC). (Lauraceae)

#### Distribution.

**China**: Guangxi, Taiwan, Yunnan, Zhejiang (Yang 1984; Yang 1995); **Japan**: Ehime, Fukuoka, Gunma, Ibaraki, Kagoshima, Nagasaki, Nara, Oita, Osaka, Saga, Tochigi, Tokyo (Kuwayama 1949; Miyatake 1970; HIC, OMNH); **Nepal**: Kathmandu Valley, Mt. Phulchowki (Miyatake 1981).

#### Biology.

Miyatake (1970) elaborately recorded the biology of the species on *Lindera erythrocarpa* and *L. glauca*. The females lay scattering eggs on the adaxial side of spread young leaves, and by oviposition, pit galls which protrude on the abaxial surface are formed, each is occupied by one later molted immature. The species seems bivoltine, and overwinters as adults on ever green trees (at least in temperate areas of Japan).

### Togepsylla takahashii

Taxon classificationAnimaliaHemipteraPsylloidea

Kuwayama, 1931

Figs 3 , 7 , 11 , 15 , 18 , 25 , 26 , 31 , 38–42 , 47–51 , 53 , 56 , 57 , 59

Togepsylla takahashii Kuwayama, 1931: 121; Takahashi 1936: 292; Yang 1984: 188; Li 2011: 213.
Togepsylla minana Yang & Li, 1981: 179. Synonymized by Li 2011: 213.

#### Diagnosis.

Fore wing with yellow bands (Fig. 11). Metabasitarsus with a pair of thickened setae on apex (Fig. 15). Paramere with a sclerotized tooth anteriorly (Figs 25, 26). Female proctiger long and smoothly tapering apically (Fig. 31).

#### Redescription.

#### Adult coloration.

Ground color yellow. Compound eyes grey. Long and thick setae on dorsum black. Ocelli yellow. Antennae yellow, with black spices on segments III-VIII; segments IX-X entirely black. Fore wing hyaline, with four obliquely transverse yellow stripes (Fig. 11). Legs yellow. Abdominal tergites brown. Male and female terminalia yellow.

Structures: Setae on dorsum of body relatively long (Table 1) and based on prominent projections. Torulus produced and slightly turned outwards (Fig. 3). Gena flat (Fig. 3). Antennal segments III-IX each with a single rhinarium on the apex, the ones on segments V and VII with small horn-shaped projections; proximally based terminal seta slightly longer than the distally based one (Fig. 7).

Mesoscutum with four pairs of prickly setae (Fig. 53). Metatibia with two rows of thick setae lateral-ventrally, and with a tightly packed row of short setae on the dorsum (Fig. 15). Apex of metabasitarsus with a pair of thick setae (Fig. 15). Pulvilli narrow (Fig. 15). Fore wing with long and narrow cell r_1_, vein M_1+2_ rather close to vein Rs, cell cu_1_ tallest in the middle; vein M_3+4_ lacking seta; surface spinules as tiny thick spines, widely spread across a large area on wing membrane; fields of radular spinules relatively large (Fig. 11).

Pore fields on abdominal ventrum long, narrow and curved; pores tightly packed (Fig. 18).

Male terminalia: Distal 1/3 of proctiger with posterior surface split and replaced with membranous tissue (Fig. 25). Paramere slender and bilobed; apex of anterior lobe developed into a sclerotized tooth; anterior margin of basal 1/3 emarginated; two long and thick setae present on inner surface, near the anterior margin; apical half with a curved vertical row of small peg setae on inner surface, near posterior margin (Fig. 25, 26). Aedeagus curved forward at apical 1/4, dorsum with a short row of spines that gradually turn smaller apically (Fig. 25). Dorsal-apical angle of subgenital plate produced and with a long seta (Fig. 25).

Female terminalia (Fig. 31): Long and straight in overall shape. Base of proctiger slight raised, apical process without tiny setae. Subgenital plate with acute apex, ventral surface with relatively dense and nearly evenly spaced setae.

***Fifth instar immature.*** Body dorsum firmly sclerotized, with sclerites of thorax and abdomen almost unseparated; body ventrum weakly sclerotized. Dorsum of head, thorax, and abdomen with symmetrical acute sectasetae varying in size (Fig. 38); dorsum and margin of wing pads with roughly symmetrical acute sectasetae (Fig. 40). Head with 1+1 bulges, sheathing the central two pairs of long setae of adult head (Fig. 38); 1+1 projections present before fore wing pads, sheathing the 2+2 long setae on lateral margins of adult pronotum (Fig. 40). Antennae 9-segmented, apices of segments 5, 7 and 8 each with one single rhinarium (Fig. 39). Compound eyes with 1+1 ocular acute sectasetae, postocular acute sectasetae present in 2+2 (Fig. 38). Fore wing pad with two pores on dorsum (Fig. 40). Tarsal pulvilli narrow (Fig. 41). Abdominal ventrum with 5 pairs of spiracles surrounded by peritremes fused with central sclerites. Abdominal apex produced as a small pair of rounded bulges (Fig. 42). Anus terminal, circum anal rings present both dorsally and ventrally. Outer circum anal ring composed of oval pores, significantly expanded bilaterally, anterior aspect strongly crooked; inner circum anal ring composed of minute oval pores, expanded bilaterally, single rowed in the middle (Fig. 42).

**Figure 47–53.**
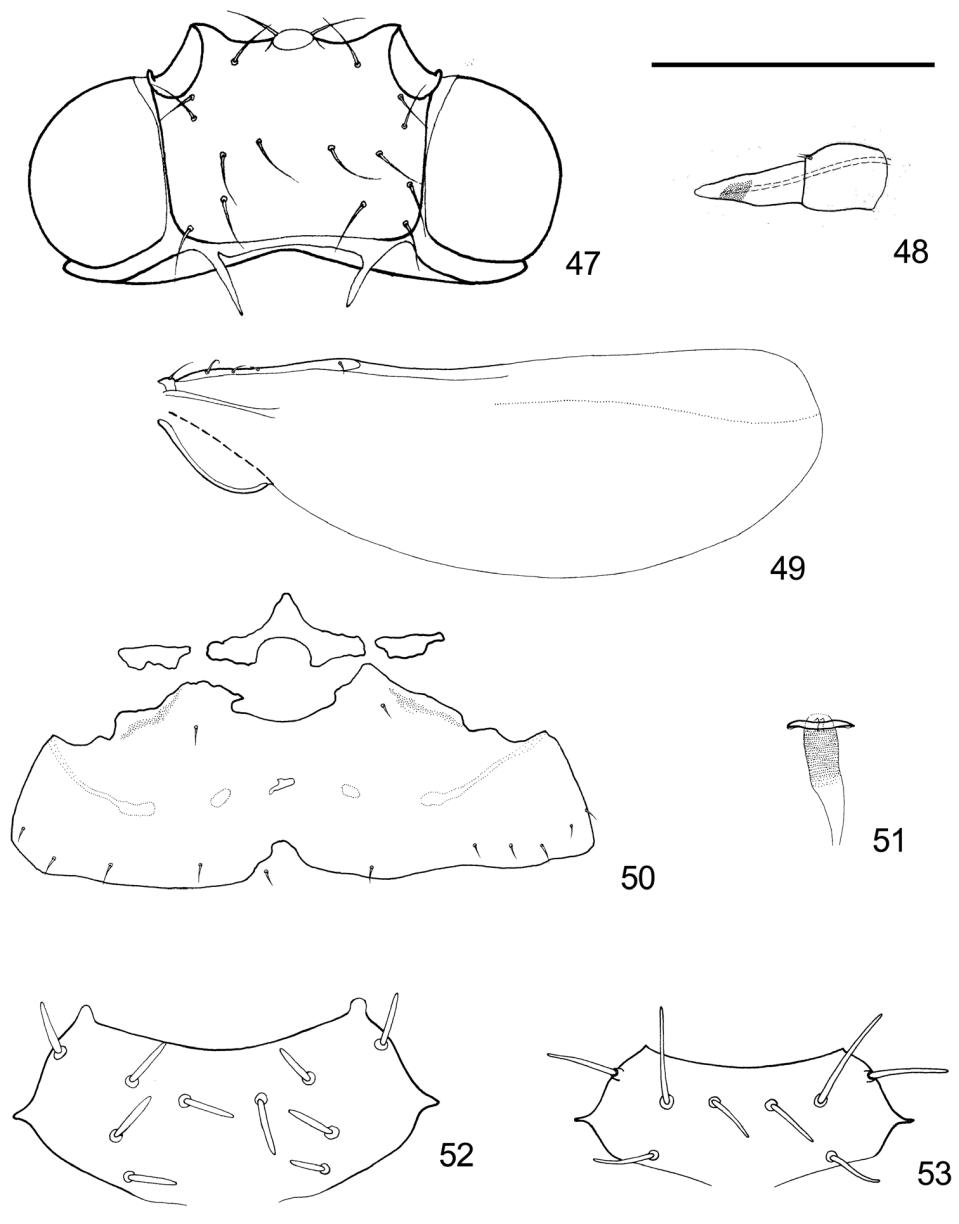
Various parts of *Togepsylla* spp. **47–51, 53** *Togepsylla takahashii* **52** *Togepsylla matsumurana* **47** Head, ventral view **48** Labium **49** Hind wing **50** Tergites of abdominal segments 1–3 **51** Sperm pump. Scale bar: 0.2 mm (**47, 48, 50, 51**), 0.5 mm (**49**), 0.32 mm (**52, 53**).

**Figure 54–56.**
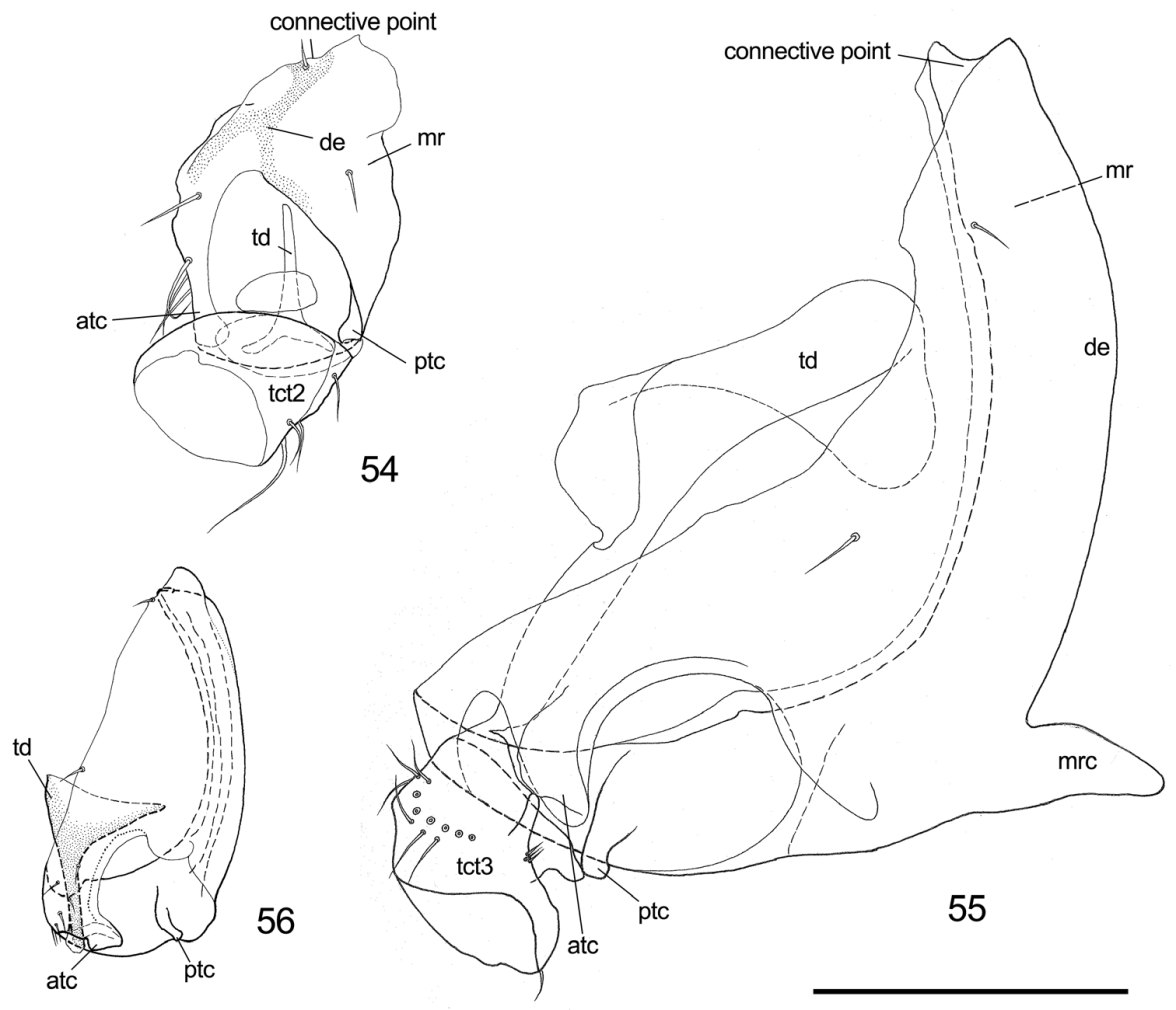
Comparison of coxa of different psyllid taxa. **54, 55** *Cacopsylla* sp. **56** *Togepsylla takahashii* **54** Mesocoxa and trochanter **55** Metacoxa and trochanter; 56. Metacoxa. Scale bar: 0.2 mm. Abbrevations: atc = anterior trochanteral condyle; de = dorsal edge; mr = meron; mrc = meracanthus; ptc = posterior trochanteral condyle; tct = trochanter; td = trochanteral tendon.

#### Material examined.

CHINA: 18 ♂, 21 ♀, 10 fifth instar immatures, Fujian, Shaxian, ex *Lindera communis*, 1.ix.1974, Chikun Yang and Fasheng Li (CAU, type series of *Togepsylla minana*); 35 ♂, 82 ♀, Guangxi, Liuzhou, 8.vi.1984, Fasheng Li (CAU); 40 ♂, 51 ♀, Guangxi, Lingchuan, Longkou, 5.vi.1984, Fasheng Li (CAU); 10 ♂, 5 ♀, Taiwan, New Taipei, Wulai, Fushan Nature Reserve, ex *Lindera communis*, 8.vi.2013, Xinyu Luo (CAU).

#### Host plant.

*Lindera communis* Hemsl., *L. megaphylla* Hemsl. (= *L. oldhamii*) (Lauraceae) (Takahashi 1936).

#### Distribution.

**China**: Fujian, Guangxi, Taiwan.

**Biology**: Takahashi (1936) and Li (2011) recorded that the immatures of the species feed on the abaxial surface of young leaves, inducing the edge of leaves to curl downwards, forming leaf-rolling galls that harbor large amounts of the insect. The immatures also secrete wax and honey dew. Severe damages to the host can cause most of the shoots to twist and shrink.

### Togepsylla tibetana

Taxon classificationAnimaliaHemipteraPsylloidea

(Yang & Li, 1981)

Figs 4 , 8 , 12 , 27 , 28 , 32

Hemipteripsylla tibetana Yang & Li, 1981: 182; Li 2011: 209.
Togepsylla tibetana (Yang & Li): Hodkinson 1990: 716.

#### Diagnosis.

Paramere with large area of netlike grains covering the inner surface of apical half, anterior margin serrated (Figs 27, 28). Female proctiger short, curved upwards only at the tip (Fig. 32).

#### Redescription.

***Adult coloration.*** Ground color yellow. Long and thick setae on dorsum yellow. Compound eyes grey. Ocelli yellow. Antennae yellow, with black spices on segments IV, VI, VIII; segments IX-X entirely black. Fore wing hyaline and colorless (Fig. 12). Male and female terminalia yellow.

Structures: Setae on dorsum of body relatively short (Table 1) and based on smooth projections. A pair of small tubercles present above toruli (Fig. 4). Gena moderately swollen bilaterally (Fig. 4). Antennal segments IV-IX each with a single rhinarium on the apex, segments IV, VI and VIII each with an extra rhinarium; all rhinaria with horn-shaped projections; proximally based terminal seta about equally long with the distally based one (Fig. 8).

Mesoscutum with five pairs of prickly setae. Metatibia with one row of thick setae ventrally, and with a tightly packed row of long setae on the dorsum. Pulvilli narrow. Fore wing with broad cell r_1_, cell cu_1_ tallest in the middle; vein M_3+4_ with one seta on the base; surface spinules rather minute, widely spread across a large area in distal cells; fields of radular spinules unclear (Fig. 12).

Pore fields on abdominal ventrum large oval, with pores loosely packed.

Male terminalia: Proctiger completely sealed, with apex slightly thickened (Fig. 27). Paramere broad lamellar, with rather slender base; anterior margin of apical half emarginated, thin and serated; posterior margin with a basal ridge; apical half of inner surface with netlike grains; anterior angle with a few short and thick setae on inner surface; posterior margin with a row of inner-curved short setae on apical half (Figs 27, 28). Aedeagus curved backwards apically, dorsum lacking tiny spines, tip forming a small acute hook (Fig. 27). Subgenital plate near rectangular in profile, dorsal-apical angle with one long seta, ventral surface with sparse setae (Fig. 27).

Female terminalia (Fig. 32): Short and broad in overall shape. Apex of proctiger moderately curved upwards; apical half of proctiger with nearly evenly spaced setae, and with a row of setae along ventral margin of apical process. Subgenital plate with blunt and retracted apex, ventral surface with sparse setae.

**Figure 57–58.**
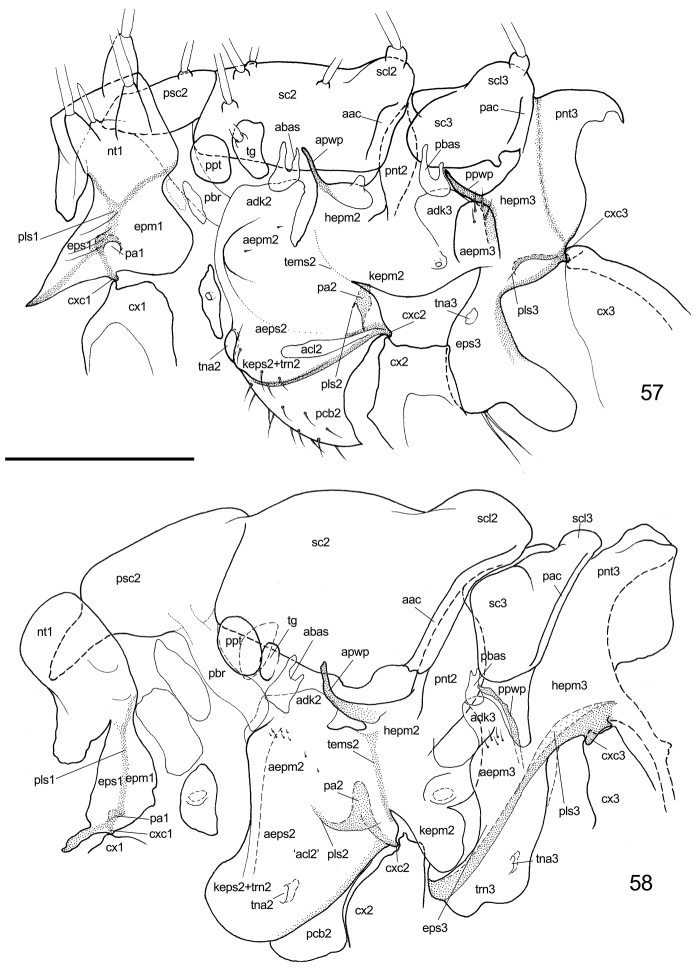
Lateral aspect of thorax. **57** *Togepsylla takahashii* **58** *Cacopsylla* sp. Scale bar: 0.2 mm (**57**), 0.5 mm (**58**). Abbrevations: aac = anterior axillary cord; abas = anterior basalare sclerite; acl = anapleural cleft; adk = anepimeral disk; aepm = anepimeron; aeps = anepisternum; apwp = anterior pleural wing process; cx = coxa; cxc = coxal condyle; epm = epimeron; eps = episternum; hepm = heel of epimeron; kepm = katepimeron; keps = katepisternum; nt = notum; pa = pleural apophysis; pac = posterior axillary cord; pbas = posterior basalare sclerite; pcb = precoxal bridge; pbr = prealar bridge; pls = pleural suture; pnt = postnotum; ppt = parapteron; ppwp = posterior pleural wing process; psc = praescutum; sc = scutum; scl = scutellum; tems = transepimeral suture; tg = tegula; trn = trochantin.

**Figure 59–61.**
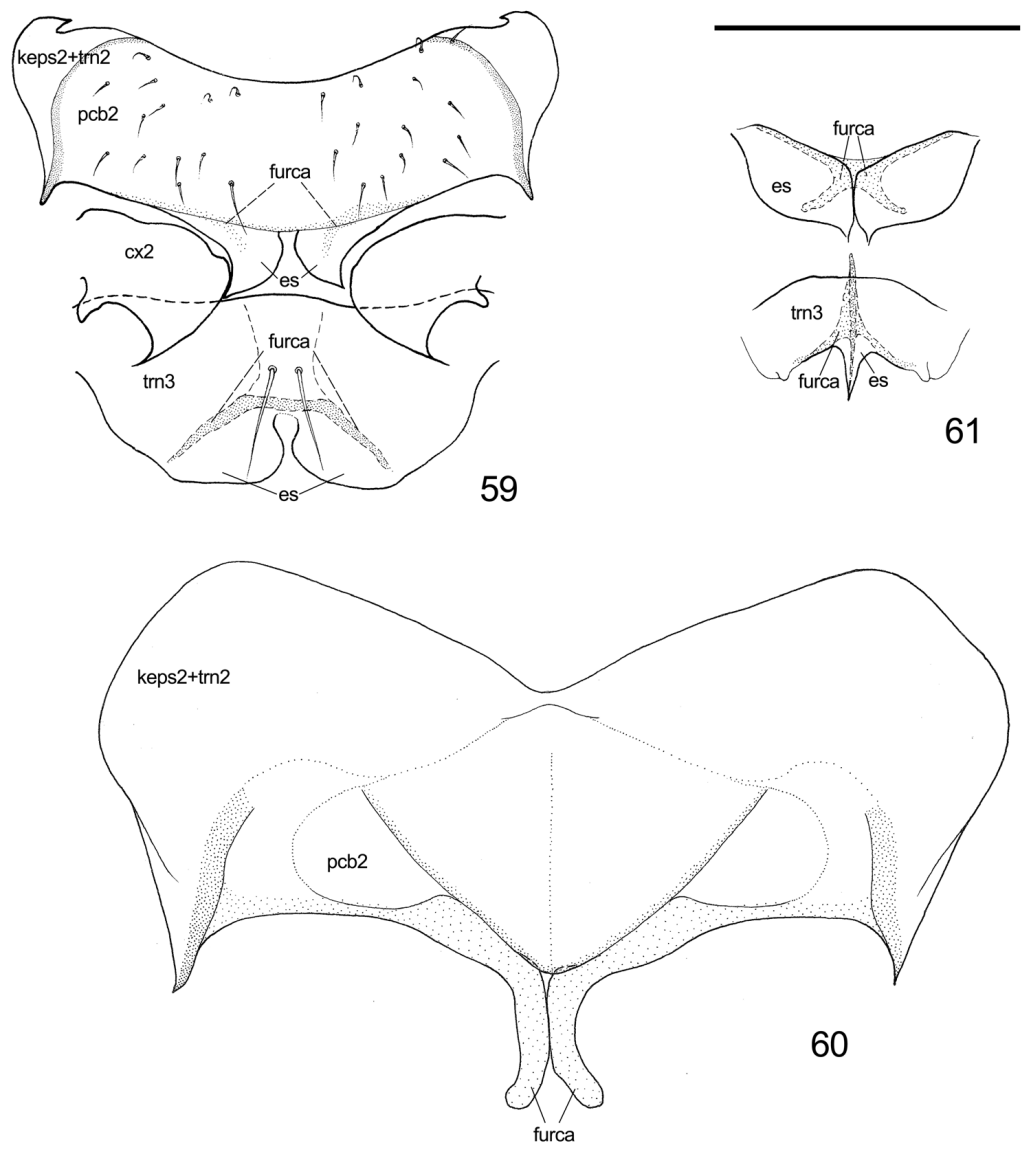
Comparison of ventral aspect of thorax. **59** *Togepsylla takahashii*, ventral aspect of meso- and metathorax **60** *Cacopsylla* sp., ventral aspect of mesothorax **61** *Trialeurodes vaporariorum*, ventral aspect of meso- and metathorax. Scale bar: 0.2 mm. Abbrevation: es = extra sclerites.

**Figure 62–63.**
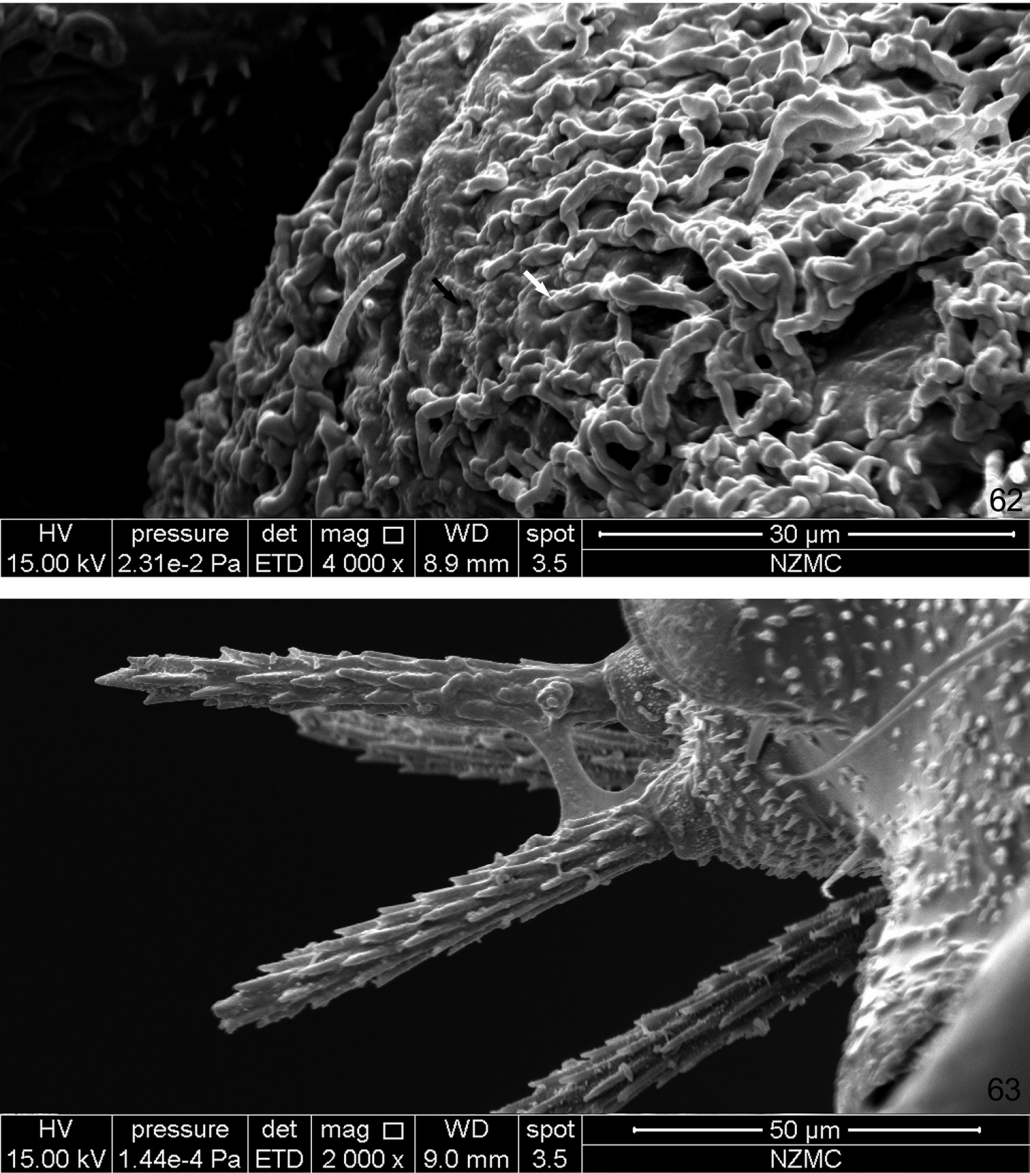
SEM photographs of *Togepsylla matsumurana*. **62** Wax-secreting field on the sternite of abdominal segment 5, black arrow showing empty pore, white arrow showing wax thread secreted **63** Prickly setae on the vertex, showing detailed structure.

***Fifth instar immature.*** Unknown.

#### Material examined.

CHINA: 49 ♂, 69 ♀, Tibet, Nyingchi, Mafenggou, 3050 m, ex *Litsea sericea*, 1.vi.1978, Fasheng Li (CAU, type series).

#### Host plant.

*Litsea sericea* (Nees.) Hook. f. (Lauraceae)

#### Distribution.

**China**: Tibet.

#### Biology.

Yang and Li (1981) recorded that the adults gather among the clusters of young leaves by large amount. The record of a habit similar with *T. takahashii* by Li (2011) seems artificial.

##### Differences between *Togepsylla* and *Syncoptozus*

The similarities and differences of the two genera have been listed by Hodkinson (1990). Nevertheless, some supplements can still be made here. *Togepsylla* possesses no median suture or discal foveae on the vertex; while Syncoptozus has the anterior section of median suture present, and *S. bifurcatus* possesses discal foveae (Brown and Hodkinson 1988). *Togepsylla* has rhinaria on antennal segments IV-IX, sometimes even segment III, and often with additional rhinaria; *Syncoptozus* has only one rhinarium on apex of segments IV, VI, VIII, and IX each.

##### Reassessment of morphology

**Hind legs**

Psyllids jump powerfully, then cast a mid-air rotation. Such a somersault, however, involves not only the strong muscles supported by the specialized metathoracic furca, enlarged metatrochanteral tendon and expanded meral part of the metacoxa but also a kicking of both hind legs on parallel planes (Burrows 2012), which are also parallel to the longitudinal body axis. This longitudinal placement of hind legs is caused by an inward twist of the metacoxa.

To discuss the formation of the enlarged and twisted metacoxa, one must seek reference from the mesocoxa. Mid and hind legs are both appendages of winged thoracic segments; additionally, in immature psyllids, they are equal in every detail, although differing from the forelegs in some aspects, indicating that hind legs of adults emerged from the model of mid legs. An undescribed *Cacopsylla* species is used as example:

The mesocoxa (Fig. 54) are relatively small and are connected to the coxal condyle of the mesopleurite by a dorsal-most articulation. Starting from the articulation, a thickened vertical edge runs down the outer surface, facing the lateral aspect, and is termed here as the ‘dorsal edge’ of the coxa. The coxa connects to the trochanter via two ‘trochanteral condyles’, which are longitudinally positioned, thus respectively termed ‘anterior-’ and ‘posterior trochanteral condyle’. Such longitudinal positioning of trochanteral condyles places the mid legs on a transverse plane, a plane nearly perpendicular to the longitudinal body axis. Besides, a normally developed trochanteral tendon originates on the inner-dorsal edge of the mesotrochanter, stretching into the chamber of the mesocoxa, clinging onto the corresponding muscles.

Compared with mesocoxa, the metacoxa (Fig. 55) first experienced an enlargement of the coxal wall, which pivots over the elongation of the dorsal edge and is primarily characterized as the expansion of the prearticular part of the coxal wall and thickening of the meron. Simultaneously, because of the unequal development of the prearticular part and the meron, the entire metacoxa is twisted backwards at approximately 90°, turning the two trochanteral condyles into a transverse position. The plane of hind leg is therefore turned longitudinal (Figs 66, 67). This pair of straightly backwards-reaching hind legs provides a much better concentration of jumping force, thereby driving the powerful jump described above. Additionally, the trochanteral tendon is magnified and possesses a tortuous apex, serving to support the strong jumping muscles.

**Figure 64–67.**
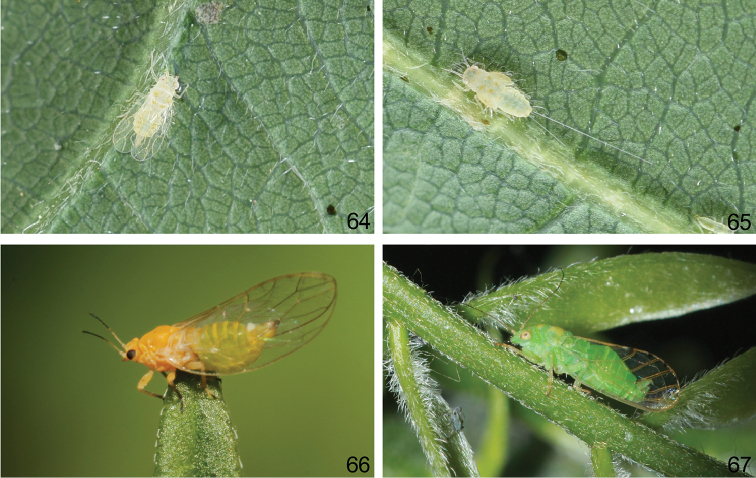
Habitus, showing difference in the ways that hind legs are held. **64** *Togepsylla glutinosae* sp. n., adult **65** *Togepsylla glutinosae* sp. n., immature **66** *Trioza urticae* (Linnaeus), adult **67** *Cyamophila hexastigma* (Horvath), adult.

By contrast, *Togepsylla* possesses half-modified metacoxae (Fig. 56). The enlargement is almost complete, but the positioning of the two trochanteral condyles is shifted at a limited level. For this reason, the hind legs of *Togepsylla* retain a posture similar to that of the middle legs, as shown in the habitus photograph (Fig. 64). Additionally, the trochanteral tendon is also half-enlarged: the relative size is much smaller, and the apex, although also expanded, is a simple flat surface instead of tortuous. According to the field observations by Xinyu Luo, adults of *Togepsylla glutinosae* sp. n. can only leap forward like frogs, at a short distance and without mid-air rotations.

**Lateral aspect of thorax**

Most psyllids possess an apophysis on meso- and metepisternal complex, termed ‘trochantinal apodeme’ (Ouvrard et al. 2002). This is an autapomorphy of Psylloidea. For mesopleuron, this structure may be on the anterior margin or median portion, depending on the taxon (Ouvrard et al. 2002). However, there are some cases like *Togepsylla* and *Pseudophacopteron* in which the trochantinal apodeme is placed on the anterior margin and reduced to an obscure vestige.

According to Ouvrard et al. (2002), the modification of psyllid metapleurite relative to mesopleurite is due to a curving of the pleural sulcus. For the metapleurite of most psyllids, taking *Cacopsylla* as example, the pleural sulcus turns downwards over the coxal condyle, becoming congruent with the elongated and internally ridged dividing suture of episternum and trochantin (Fig. 58). In *Togepsylla*, the metapleuron represents a halfway modification. The dividing suture of metepisternum and trochantin is absent, the trochantinal apodeme is shallow and in anterior position, as in mesothorax (Fig. 57).

**Wax-secreting fields on abdominal sternites**

*Togepsylla* possesses three pairs of fields of pores on sternites of abdominal segments 4–6, in both sexes. Wax secretions from these pores have been observed on *T. matsumurana* (Fig. 62). Similar structures, several pairs of wax plates composed of many small wax-secreting pores, is one of the defining characters of adult whiteflies: Whiteflies kick the wax secretions of these glands with the hind legs, and then spread the shattered wax particles over the entire body surface (Byrne and Bellows 1991). In females of Aleyrodinae, two pairs of wax plates are found, on segments 3–4, whereas in Aleurodicinae, four, on segments 3–6; in males of Aleyrodinae, four pairs are present on segments 3–6, whereas three pairs appear on segments 3–5 in Aleurodicinae and Udamoselinae (Gill 1990; Martin 2007).

All the four members of Sternorrhyncha are known to secrete wax through integumental wax gland/pores. In scale insects whose wax glands are studied the most, these structures are highly variable in ultrastructure (shape and number of loculars of each pore) and distribution (all over the body or restricted to a certain region) (Foldi and Pearce 1985; Foldi and Lambdin 1995). Some aphid families/subfamilies possess wax gland plates, which also vary in shape and distribution, on body dorsum (Chen and Qiao 2012). These, however, are not so far known to reveal the same arrangement as Togepsyllinae and whiteflies, nor does the lack of detailed ultrastructural study of Togepsyllinae support their resemblance.

Psyllid immatures possess wax-secreting pores on their caudal plates. These pores are arranged in various patterns, mostly with a basic circum-anal ring (possibly homologous with the circum-anal ring of female adults), and on many occasions with extra pore fields (Brown and Hodlinson 1985). Extra pore fields can sometimes be succeeded by the adults, appearing on their more terminal (usually segments 7 and/or 8) abdominal tergites, e.g. *Agonoscena pegani* Loginova, 1960 and *A. sabulisa* Li, 1994 (in Li et al. 1994) (Luo 2016). Although it is not currently possible to accurately decide the homology between abdominal segments between immatures and adults, one can still roughly judge and count the separate segments of immatures by the dorsal and ventral setae rows. So far, the immature of not any species possess wax secreting pores on areas that are possibly homologous with abdominal sternites 4–6.

This is the first time that a psyllid adult is found with such fields of wax-secreting pores. Compared with those of whiteflies, wax pore fields of *Togepsylla* are strongly constricted, and the segment correspondence is different. It is uncertain whether these structures of *Togepsylla* and Aleyrodoidea are homologous or not.

## Discussion

Togepsyllinae displays great differences with all the other psyllid taxa in external morphology. These include: frons completely fused with gena; gena firmly compact instead of being bisected; ‘conical sensoria’ absent from apex of labium; metapleuron distinctively arranged; metacoxa ventral aspect of metathorax as a compact sclerite; wax plates present on sternites of abdominal segments 4–6; male terminalia oriented caudally; male proctiger completely enveloped, instead of having a basal major part, which is sclerotized anteriorly and laterally, whereas membranous posteriorly, with a median suture; aedeagus one-segmented; sperm pump with only basal end plate, lacking the apical end plate; median valve of female terminalia simple, slender and placed more terminal, apex touching the subapex of ovipositor; fifth instar immatures without tarsal arolium, instead with pulvilli on claws. These traits make the current systematic position of Togepsyllinae doubtful.

Alternatively, Togepsyllinae share many similar characters with fossil pan-psyllids [extinct taxa included in Psyllomorpha by Bekker-Migdisova (1985), namely Pincombeidae, Protopsyllidiidae, Liadopsyllidae, Malmopsyllidae and Neopsylloididae] and whiteflies. With fossil pan-psyllids, the similarities include the half-modified jumping hind legs (compared with the none-jumping hind legs of Liadopsyllidae and Malmopsyllidae) (Ouvrard et al. 2010), the one-segmented aedeagus (particularly *Syncoptozus*, compared with *Postopsyllidium*) (Grimaldi 2003), frons fused with gena (compared with *Postopsyllidium*) (Grimaldi 2003). With whiteflies, the major similarities include the frons fused with gena, the pair of extra sclerites posterior to base of thoracic furcae (Fig. 61), presence of wax plates, one-segmented aedeagus, and absence of flag lobe on apex of valvula dorsalis of ovipositor. Similarities and differences among whiteflies, fossil pan-psyllids, Togepsyllinae and other modern psyllids, are listed in Table 2.

**Table 2.** Comparison of characters among whiteflies, fossil pan-psyllids, Togepsyllinae, and other psyllids *sensu stricto.*

	Aleyrodoidea	Protopsyllidae	Liadopsyllidae	Togepsyllinae	Other Psylloidea *sensu stricto*
Median suture of vertex	Absent	Absent	Absent	Absent (*Togepsylla*) or present in the anterior half of vertex (*Syncoptozus*)	Present (with a few exceptions such as *Pseudophacopteron* and *Atmetocranium*)
Frons	Completely fused with gena	Completely fused with gena	Independent from gena	Completely fused with gena	Independent from gena
Clypeus	Fused with gena	Fused with gena	Attached to gena by a pair of sclerites	Attached to gena by a pair of sclerites	Attached to gena by a pair of sclerites
Labium	Long, originated before prosternum	Long, originated before ventrum of prothorax	Long, originated between procoxae	Shortened (two-segmented), originated between procoxae	Shortened (pseudo-three-segmented), originated between procoxae
Extra sclerites posterior to base of thoracic furca	Present	-	-	Present	Usually absent, but present in Rhinocolinae
Modification of metapleurite	-	-	-	Incomplete	Complete
Modification of metacoxa	Slight enlargement	Slight enlargement	Slight enlargement	Significant enlargement, slight backwards twist	Significant enlargement, backwards-twisted at 90°
Enlargement of trochanteral tendon	None	-	-	Slight	Significant
Reduction of tergite of abdominal segment 1	Tergite complete	-	-	Consistent in the middle	Reduced to two separate small lateral sclerites
Wax plates	Present	-	-	Present	Absent
Aedeagus	One-segmented	One-segmented	-	One-segmented	Double-segmented
Male proctiger	Fused with subgenital plate	Fused with subgenital plate	-	Posterior aspect completely sclerotized and finely enveloped	Posterior aspect membranized
Valvulae dorsales of ovipositor	Without flag lobe	-	-	Without flag lobe	With flag lobe (except for *Apsylla*)
Ocular setae of last instar immature	Absent	-	-	Present	Present or absent
Tarsal arolium of last instar immature	Absent	-	-	Absent	Present

In the schematic phylogenetic tree (Burckhardt and Ouvrard 2012), Rhinocolinae, Spondyliaspidinae and Togepsyllinae were treated as sister groups; and Drohojowska (2015), using thoracic characters, also produced a phylogeny that assigned Togepsyllinae and Rhinocolinae as sister groups. The supportive characters of Drohojowska include: anapleural cleft hardly visible; ventral view of anterior protruding of katepiternum small and oval; meracanthus absent or as very small tubercle. In Rhinocolinae, the most similar with Togepsyllinae was *Apsylla*, a genus (monotypic) with completely unmodified metacoxa (Ouvrard and Burckhardt 2010). Judging from the SEM photograph given by Drohojowska (2015: Fig. 13), hind legs of *Apsylla cistellata* (Buckton) follow the same model with *Togepsylla*, being on a plane nearly parallel with that of the middle legs. While according to the illustration in Mathur (1975), *A. cistellata* possesses no flag lobe on the apex of valvula dorsalis.

In addition to the obvious synapomorphies of Aphalarinae members, i.e., mesothoracic trochantinal apodeme present on the anterior margin of the pleurite and metatibia with an open crown of apical spurs, Togepsyllinae and Rhinocolinae share other characters. They both have: a short clypeus; a pair of extra sclerites posterior to the base of thoracic furca; meracanthus absent or rather small; and the tubercle above the apical opening of metacoxa prominent. Most notable is the extra pair of sclerites posterior to the base of thoracic furcae. These shared characters may suggest a relatively close relationship between Togepsyllinae and Aphalaridae-Rhinocolinae.

Another species, *Atmetocranium myersi* (Ferris and Klyver) (Calophyidae: Atmetocraniinae), the sole member of the genus, is somewhat in resemblance with Togepsyllinae. Referring to a dry-mounted specimen and to the original description (Ferris and Klyver 1932: Fig. 15K), it was found that the species possesses the same type of hind legs as Togepsyllinae. This species also lacks the median suture on the vertex, flag lobes on valvulae dorsales of ovipositor, meracanthus on metacoxa, and metabasitarsal spurs. The immatures also possess two-segmented tarsi and lacks a tarsal arolium (Tuthill 1952). However, the male terminalia of *A. myersi* is of the common type, with proctiger and parameres oriented upward and the aedeagus two-segmented. Additionally, the gena is bisected as normal. Burckhardt and Ouvrard’s (2012) assignment of *Atmetocranium* into the higher Calophyidae was ‘provisional’, primarily based on the internal comb of apical spurs on metatibia and the one-segmented, asymmetrical antennal flagellum of immatures, but the family actually lacks defining synapomorphies (Burckhardt and Ouvrard 2012). Unfortunately, *Atmetocranium* is too scarce, and we did not have access to the slide-mounted specimens; thus, we do not know the details of its morphology, particularly those concerning the thorax. Therefore, currently, the relationship between Togepsyllinae and *Atmetocranium* remains uncertain.

Phylogeny of Togepsyllinae seems unsolvable in the current situation, given the clear fact that there are only two known genera which are distinct from each other in many traits, indicating the possible existence of further extinct members of the group. On a greater scale, the current definition of Aphalaridae needs a phylogeny-based revision, to resolve its internal relationships and to test if Togepsyllinae is an independent taxon.

## Supplementary Material

XML Treatment for Togepsylla

XML Treatment for Togepsylla glutinosae

XML Treatment for Togepsylla matsumurana

XML Treatment for Togepsylla takahashii

XML Treatment for Togepsylla tibetana
